# Disruption of O-GlcNAc Cycling in *C. elegans* Perturbs Nucleotide Sugar Pools and Complex Glycans

**DOI:** 10.3389/fendo.2014.00197

**Published:** 2014-11-24

**Authors:** Salil K. Ghosh, Michelle R. Bond, Dona C. Love, G. Gilbert Ashwell, Michael W. Krause, John A. Hanover

**Affiliations:** ^1^Laboratory of Cell and Molecular Biology, National Institute of Diabetes and Digestive and Kidney Diseases, National Institutes of Health, Bethesda, MD, USA; ^2^Laboratory of Molecular Biology, National Institute of Diabetes and Digestive and Kidney Diseases, National Institutes of Health, Bethesda, MD, USA

**Keywords:** O-GlcNAcylation, nucleotide sugars, hexosamines, *C. elegans*/nematode, glycogen, trehalose

## Abstract

The carbohydrate modification of serine and threonine residues with O-linked beta- *N*-acetylglucosamine (O-GlcNAc) is ubiquitous and governs cellular processes ranging from cell signaling to apoptosis. The O-GlcNAc modification along with other carbohydrate modifications, including N-linked and O-linked glycans, glycolipids, and sugar polymers, all require the use of the nucleotide sugar UDP-GlcNAc, the end product of the hexosamine biosynthetic pathway (HBP). In this paper, we describe the biochemical consequences resulting from perturbation of the O-GlcNAc pathway in *C. elegans* lacking O-GlcNAc transferase and O-GlcNAcase activities. In *ogt-1* null animals, steady-state levels of UDP-GlcNAc/UDP-GalNAc and UDP-glucose were substantially elevated. Transcripts of genes encoding for key members in the HBP (*gfat-2, gna-2, C36A4.4*) and trehalose metabolism (*tre-1*, *tre-2*, *tps-2*) were elevated in *ogt-1* null animals. While there is no evidence to suggest changes in the profile of N-linked glycans in the *ogt-1* and *oga-1* mutants, glycans insensitive to PNGase digestion (including O-linked glycans, glycolipids, and glycopolymers) were altered in these strains. Our data support that changes in O-GlcNAcylation alters nucleotide sugar production, overall glycan composition, and transcription of genes encoding glycan processing enzymes. These data along with our previous findings that disruption in O-GlcNAc cycling alters macronutrient storage underscores the noteworthy influence this posttranslational modification plays in nutrient sensing.

## Introduction

Posttranslational modifications ranging from glycosylation to phosphorylation play critical roles in biological processes including protein localization, transcription, and cellular signaling [see Ref. (1) and references therein]. The posttranslational modification O-linked beta-*N*-acetyl glucosamine (O-GlcNAc) is ubiquitous throughout the nucleus and cytoplasm modifying over 4000 protein substrates including nuclear pore and transcription complexes, proteasomes, and kinases (2). The addition and removal of O-GlcNAc to serine and threonine residues is governed by two enzymes, O-GlcNAc transferase (OGT) and O-GlcNAcase (OGA), respectively (3). This dynamic cycling occurs faster than protein turnover poising O-GlcNAc to act as a signaling molecule (4).

UDP-GlcNAc, the activated nucleotide sugar utilized by OGT, is the end product of the hexosamine biosynthetic pathway (HBP). A metabolite responsible for sensing the nutrient status of the cell, UDP-GlcNAc is synthesized by a series of enzymes utilizing key metabolites including glucose, l-glutamine, acetyl-CoA, and UTP (5). Perturbation of the HBP is linked with modulation in insulin signaling and glucose toxicity [see Ref. (6) and references therein]. Indeed, globally decreased levels of UDP-GlcNAc can be profoundly damaging to mammals (7). The way in which the HBP plays a role in the modulation of insulin signaling and glucose toxicity is currently being defined with implications that O-GlcNAcylation plays an important mechanistic role [see Ref. (8) and references therein].

We previously demonstrated that *C. elegans* loss-of-function *ogt-1* and *oga-1* animals have altered insulin signaling and carbohydrate–fat metabolism (9). Importantly, *ogt-1* and *oga-1* null *C. elegans* are viable while loss of OGT and OGA activity in higher eukaryotes yields embryonic lethality (10, 11). *C. elegans* lacking OGT-1 activity exhibit no addition of O-GlcNAc to protein serine and threonine residues and animals without OGA-1 activity lack the capacity to remove the modification. In order to better understand the repercussions of impaired O-GlcNAc cycling in a whole organism, we chose to define the way in which these perturbations altered UDP-GlcNAc concentrations, glycan composition, and transcription of important metabolic enzymes. Among the most striking differences, we found that animals lacking OGT-1 or OGA-1 activities exhibit increases in UDP-HexNAc pools and differences in their overall glycan compositions compared to wild type (N2). Indeed, along with these changes, *ogt-1* null animals also show increased transcription of metabolic and HBP genes suggesting that the animals are modulating or attempting to compensate for the altered carbohydrate profiles. Although O-GlcNAc cycling has been linked to nutrient sensing, we provide the first evidence in a whole organism that O-GlcNAc plays an important role in modulating nucleotide sugar utilization and the steady-state levels of transcripts encoding key HBP enzymes. We suggest that O-GlcNAc acts as a rheostat to fine-tune evolutionarily conserved components of *C. elegans* metabolism giving a defined model to study how changes in O-GlcNAc may impact human health.

## Materials and Methods

### *C. elegans* and bacterial strains and maintenance

The following *C. elegans* strains were used in this study: N2 Bristol (WT), *ogt-1 (ok430), ogt-1 (tm1046)*, and *oga-1 (ok1207)*. The *ogt-1 (ok430)* and *oga-1 (ok1207)* strains were provided by the *C. elegans* Gene Knockout Consortium (CGC, Oklahoma Medical Research Foundation, Oklahoma City) and the *ogt-1 (tm1046)* was provided by the National Bioresource Project of Japan. All strains were backcrossed four times to N2 prior to use in experiments. The presence of the deletion alleles was confirmed by nested PCR primers as described by the CGC. The OP50 *E. coli* was cultured without antibiotic at room temperature in Luria–Bertani (LB) broth and plated on nematode growth media (NGM) plates or 2% agarose-topped LB plates. NGM plates were made with tryptone rather than peptone. *C. elegans* were maintained on NGM plates supplemented with OP50 *E. coli* and animals were manipulated using standard techniques (12, 13). Two percent agarose-topped LB plates were used for RNA isolation experiments only.

### Isolation of nucleotide sugar

*C. elegans* strains were grown in large quantities starting from synchronous L1 stage and collected when animals reached the gravid adult stage (after approximately 72 h incubation at room temperature, 22°C). Animals were washed from NGM plates with water, washed twice with water, counted, and purged by rocking animals for 30 min in water at room temperature as previously described (9). Animals were isolated by centrifugation and the supernatant removed first by pipette and then by lyophilization. To 8 mg lyophilized worms, 0.75 ml cold 0.5 N perchloric acid was added. The suspension was vortexed vigorously for 20 s in an ice bath followed by centrifugation at 15,000 × *g* for 10 min at 4°C and the supernatant was collected. The remaining pellet was extracted second time and the two supernatants was pooled. Two hundred microliters of charcoal suspension (30 mg of Mallinckrodt charcoal/ml of 1 N perchloric acid) was added to the cold supernatant and stirred vigorously in an ice bath. The activated charcoal in acidic solution binds the nucleotide sugar and the sugars are then released in the alkaline solution in the following steps. After centrifugation at 15,000 × *g* for 10 min at 4°C, the supernatant was discarded. The charcoal pellet was eluted three times with 750 μL of a solution containing 50% ethanol + 1% NH_4_OH. The EtOH solutions were isolated, pooled, frozen, and lyophilized. This experiment was performed in triplicate. Isolated sugars were analyzed by high performance anion exchange chromatography (HPAEC) with pulse amperometric detector (PAD) using a PA10 anion exchange column as described by Suriano et al. (14). Error bars represent SD of an experiment done in triplicate and *P* values were calculated by an unpaired Student’s *t*-test. ns: *P* > 0.05; *P < 0.05 compared to N2.

### Isolation of PNGase-sensitive glycans

Glycoprotein rich fractions were isolated from *C. elegans* at synchronous L4 larval stage by procedures described by Cipollo et al. (15, 16). Further processing was analogous to preparation described in the aforementioned papers with minor modifications. Isolated glycoproteins were treated with l-1-tosylamido-2-phenylethyl chloromethyl ketone trypsin for 4 h at 37°C in 50 mM ammonium bicarbonate, pH 8.5 buffer. The reaction was stopped by boiling the samples twice for 10 min. The resulting peptides were isolated by acetone (80%) precipitation. N-glycans were released from these glycopeptides by incubating with PNGase A (Roche) and F (Prozyme) enzymes according to the manufacturers’ protocols. PNGase-sensitive glycans were pooled and purified by passing through LudgerClean™ EB10 Glycan Cleanup Cartridge following the manufacturer’s protocol. Resulting glycans were hydrolyzed with 2 M TFA for 4 h at 100°C to yield monosaccharides. Samples were evaporated to dryness and resuspended in 100 μL of deionized water. This evaporation and resuspension was repeated twice with the last 100 μL water suspension passed through a cation exchange resin to remove amino acids and peptides. A portion of this resulting monosaccharide-containing solution was profiled by HPAEC-PAD as described elsewhere (14, 17). To measure GalNAc and GlcNAc, the concentrations of monosaccharides galactosamaine (GalN) and glucosamine (GlcN) were measured by HPAEC as, immediately following their release, the *N*-acetyl sugars undergo quantitative deacetylation. Error bars represent SD of an experiment done in triplicate, and *P* values were calculated by an unpaired Student’s *t*-test. ns: *P* > 0.05; *P < 0.05; **P < 0.005; ***P < 0.0005 compared to N2.

### Isolation of RNA from L4 stage *C. elegans*

OP50 grown on agarose coated LB plates were seeded with synchronized L1 N2, *ogt-1 (ok430)*, and *oga-1 (ok1207)* strains. Animals incubated at room temperature (22°C) were collected after roughly 48 h when N2 animals reached a synchronous L4 stage. Although animals were synchronous at L1, we noticed that *ogt-1 (ok430)* animals had a difference in growth rate and we accommodated by plating them on OP50 4 h prior to the other strains. Collected L4 populations contained 97, 95, 80, and 60% L4 animals in N2, *oga-1*, *ogt-1 (tm1046)*, and *ogt-1 (ok430)* strains, respectively. The remaining animals ranged from stages L1 to L3. *C. elegans* were isolated from plates, thoroughly washed with water to remove bacteria, and worm pellets were stored −80°C for further use. RNA was isolated from each strain using a Qiagen RNAeasy Mini kit and quantified spectrophotometrically as described in Ref. (4, 18).

### qRT-PCR analysis

RNA was treated with DNAse from Invitrogen to destroy contaminating genomic DNA in RNA sample (4, 18). SuperScript III (Invitrogen) was used to produce cDNA according to manufacturer’s protocols with random primer from Promega. Following cDNA synthesis, quantitative real-time PCR (qRT-PCR) was performed using SYBR Green to quantitatively determine gene expression. RNA treated without reverse transcriptase served as the negative control. The experiment was performed in biological triplicate and samples were normalized to the control gene *act-4*. qRT-PCR data were analyzed by the comparative *C*_t_ method, error bars represent SD, and *P* values were calculated by an unpaired Student’s *t*-test. ns: *P* > 0.05; *P < 0.05; **P < 0.005; ***P < 0.0005 compared to N2.

### Gene accession information

Gene public name, Gene WormBase ID: *ogt-1*, K04G7.3, WBGene00003858; *oga-1*, T20B5.3, WBGene00020596; *gfat-2*, F22B3.4, WBGene00009035; *gna-1*, B0024.12, WBGene00001646; *gna-2*, T23G11.2, WBGene00001647; C36A4.4, WBGene00007965; *tre-1*, F57B10.7, WBGene00006607; *tps-2*, F19H8.1, WBGene00006603; *gsy-1*, Y46G5A.31, WBGene00001793; Y73B6BL.4, WBGene00022233; *act-4*, M03F4.2, WBGene00000066.

## Results

### Nucleotide sugar levels are increased in *C. elegans* ogt-1 and oga-1 mutants

**Figure 1:**
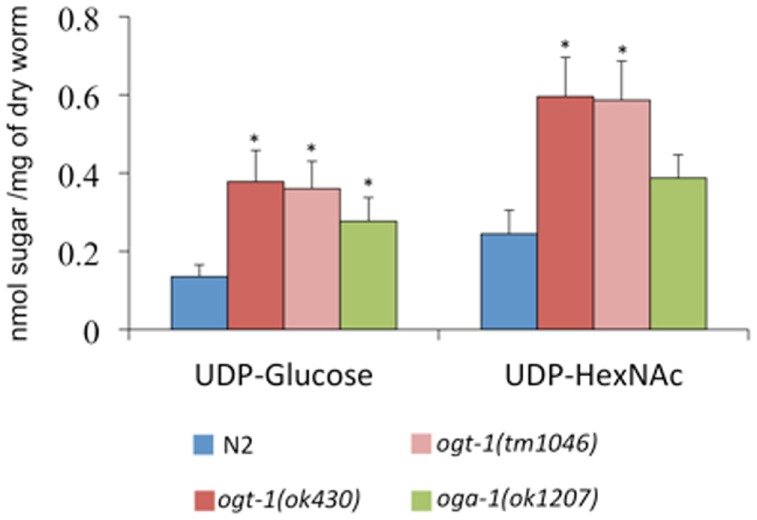
**O-GlcNAc cycling mutants exhibit increased concentrations of nucleotide sugar**. UDP-nucleotide sugars were assessed by HPAEC-PAD detection. Compared to N2 animals, *ogt-1 (ok430)*, *ogt-1 (tm1046)*, and *oga-1 (ok1207)* animals have increased levels of UDP-nucleotide sugar. Error bars represent SD of an experiment done in triplicate. ns: *P* > 0.05; *P < 0.05 compared to N2.

The *C. elegans* nucleocytoplasmic enzyme OGT-1 relies on a portion of the UDP-GlcNAc pool to glycosylate serine and threonine residues with O-GlcNAc. We sought to determine the upstream consequences when either OGT-1 activity, or the activity of its counterpart OGA-1, were absent in *C. elegans*. We initially profiled activated nucleotide sugar concentrations by isolating the nucleotide sugar pools, treating them with mild acid hydrolysis, and profiling the freed monosaccharides by HPAEC-PAD (Figure 1). We were intrigued to find that animals lacking either OGT-1 or OGA-1 activity demonstrated increased pools of UDP-glucose and *ogt-1* animals had increased UDP-GlcNAc/UDP-GalNAc (represented as UDP-HexNAc) pools. Loss of OGT-1 yielded animals with over a twofold increase in these nucleotide sugars compared to N2 while *oga-1* animals demonstrated a more modest increase. Importantly, the increased nucleotide sugar pool was similarly elevated in both *C. elegans ogt-1* null alleles that we used throughout the paper to further support our conclusions (Figure 1). Additional nucleotide sugars profiled (GDP-mannose, GDP-fucose, and UDP-galactose) were present in minimal amounts and their concentrations remained unchanged in *ogt-1 (ok430)*, *ogt-1 (tm1046)*, and *oga-1 (ok1207)* animals compared to N2 (data not shown).

### PNGase resistant monosaccharides are increased in *ogt-1* null animals

**Figure 2:**
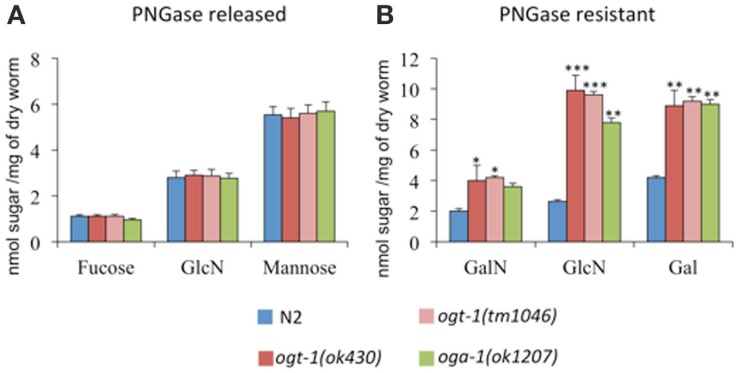
***C. elegans* lacking OGT-1 and OGA-1 activity show increased GalN, GlcN, and Gal monosaccharides compared to N2 animals (B)**. Concentrations of monosaccharides galactosamaine (GalN) and glucosamine (GlcN) were measured by HPAEC as, immediately following their release, the *N*-acetyl sugars GalNAc and GlcNAc, respectively, undergo quantitative deacetylation. No statistically different changes were observed for the composition of PNGase A and F sensitive glycans for O-GlcNAc cycling mutants when compared to N2 animals **(A)**. Error bars represent SD of an experiment done in triplicate. ns: *P* > 0.05; *P < 0.05; **P < 0.005; ***P < 0.0005 compared to N2.

UDP-GlcNAc is required for the production of a myriad of glycans, which suggests that changes in its concentration may affect glycan synthesis. Indeed, increased pools of UDP-GlcNAc have been correlated with the production of tri- and tetra-antennary N-glycans in mammary carcinoma cells (19). To determine whether complex glycan synthesis was affected by deletion of the *C. elegans* O-GlcNAc cycling enzymes, we assessed whole animal glycan composition. Briefly, isolated N-glycans were released by PNGase A and F, pooled, purified, and hydrolyzed with TFA. The resulting monosaccharide concentrations were assessed by HPAEC-PAD. Although *ogt-1* animals exhibit over a twofold increase in UDP-HexNAc levels compared to N2 (Figure 1), the released monosaccharides from N2, *ogt-1 (ok430)*, *ogt-1 (tm1046)*, and *oga-1 (ok1207)* animals’ N-glycans remain largely similar (Figure 2A). Specifically, the ratio of mannose to GlcNAc suggests N-glycans form normally in these mutants.

Glycan structures not cleaved by PNGase A and F include O-linked glycans, glycolipids, and glycopolymers. To determine whether the PNGase-insensitive glycans were altered in *ogt-1* and *oga-1* null animals, the residual glycoprotein pellets were treated with 2 M TFA and the freed sugar pools were assessed. The *ogt-1* and *oga-1* null animals exhibited up to 3.7-fold higher GalNAc, GlcNAc, and galactose levels compared to N2 worms suggesting structural changes in glycan composition for PNGase-insensitive glycans when O-GlcNAc cycling is perturbed (Figure 2B).

### Genes encoding enzymes involved in UDP-GlcNAc synthesis and metabolism are perturbed in O-GlcNAc cycling mutants

With nucleotide sugar pools and PNGase-insensitive glycan structures affected by the loss of *ogt-1* and *oga-1*, we hypothesized that the transcription of genes encoding enzymes involved in nucleotide sugar production and metabolism would be affected. To address this question, we examined the expression of key components of the HBP as well as trehalose and glycogen metabolism modules by qRT-PCR.

**Figure 3:**
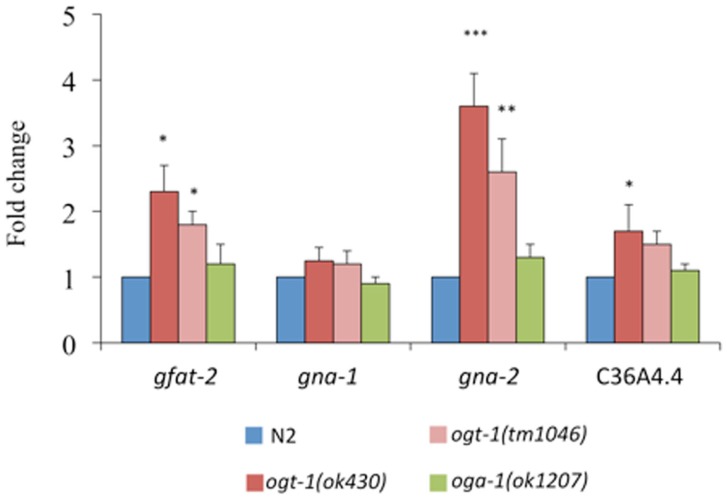
**Transcripts of genes encoding key HBP enzymes are elevated in *ogt-1* null animals**. Animals lacking OGT-1 activity show increased transcripts of *gfat-2*, *gna-2*, and C36A4.4 genes while animals lacking OGA-1 activity are not statistically different from N2. Error bars represent SD of an experiment done in triplicate. ns: *P* > 0.05; *P < 0.05; **P < 0.005; ***P < 0.0005 compared to N2.

The following genes encode key enzymes in the HBP: *gfat-2* (F22B3.4 encodes the HBP’s rate-liming glucosamine:fructose-6-phosphate aminotransferase – GFAT-2), *gna-2* (a glucosamine-6-phosphate *N*-acetyltransferase), and C36A4.4 (the putative UDP-GlcNAc pyrophosphorylase orthologous to human UAP1) (20). Transcripts for *gfat-2*, *gna-2*, and C36A4.4 are all elevated in *ogt-1 (ok430)* mutants while the transcript levels in *oga-1 (ok1207)* mutants remain unchanged (Figure 3). Elimination of OGT-1, an enzyme that utilizes a portion of the UDP-GlcNAc pool, affects the transcription of genes required for the synthesis of the same nucleotide sugar. This is not surprising as the HBP is exquisitely sensitive to nucleotide sugar concentrations and we suggest that gene transcription changes may be due to changes in feedback inhibition within the HBP. It is possible that there are no changes in HBP gene transcription for animals lacking OGA-1 activity as OGT-1 can still actively use UDP-GlcNAc.

**Figure 4:**
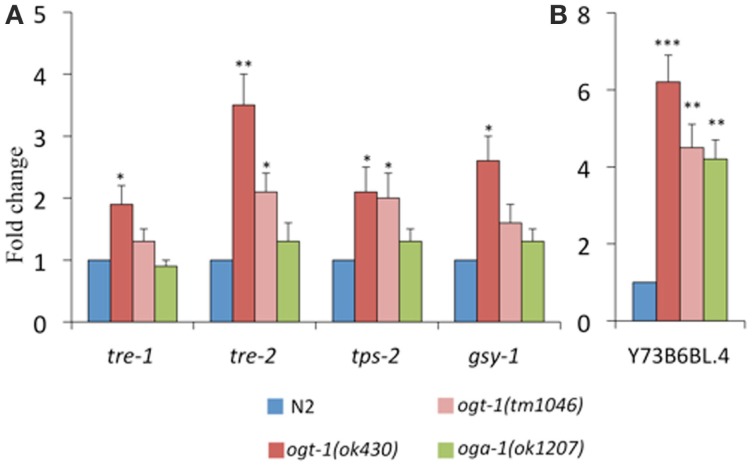
**Transcripts of genes encoding enzymes critical for metabolism are altered in *C. elegans* O-GlcNAc cycling mutants**. *ogt-1 (ok430)* animals exhibit an increases in **(A)** *tre-1, tre-2, tps-2*, *gsy-1*, and **(B)** Y73B6BL.4 while *oga-1 (ok1207)* animals show an increase in only Y73B6BL.4. Error bars represent SD of an experiment done in triplicate. ns: *P* > 0.05; *P < 0.05; **P < 0.005; ***P < 0.0005 compared to N2.

Our previous work identified that total amounts of glycogen and trehalose, two important forms of energy storage, which can be enzymatically broken down to glucose, were increased in *ogt-1* and *oga-1* null animals (9). Here, we find that in comparison to N2 animals, *ogt-1* null animals exhibit increased transcription for enzymes involved in trehalose and glycogen metabolism (*tre-1*, *tre-2*, *tps-2*, and *gsy-1*) (Figure 4A). Interestingly, while *ogt-1 (ok430)* animals exhibit nearly twofold changes for all four genes, the *oga-1 (ok1207)* animals exhibited no statistical changes from N2 suggesting that changes in the ability of the animal to add and remove the O-GlcNAc modification have different biological consequences.

Triglyceride levels are significantly altered when OGT activity is perturbed in mice and *C. elegans* (9, 10, 21). To better understand our data showing 40–70% decreases in triglyceride levels in O-GlcNAc cycling mutants (9, 10), we assessed the level of transcription of Y73B6BL.4, a gene encoding a phospholipase (22). Indeed, this phospholipase increased over fourfold for both *ogt-1* alleles and the *oga-1 (ok1207)* animals (Figure 4B).

## Discussion

**Figure 5:**
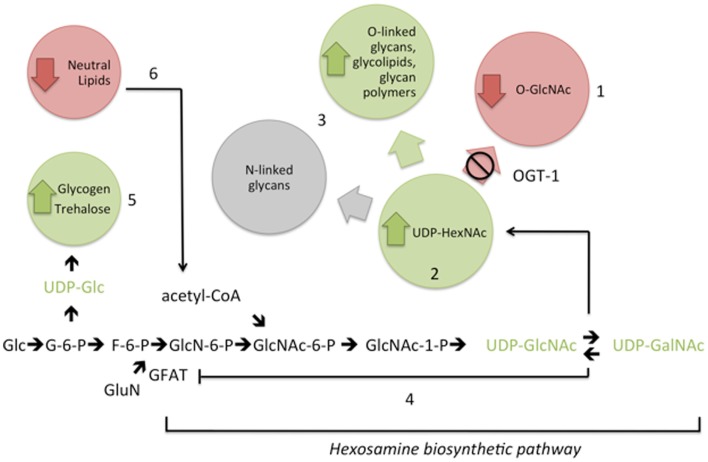
**Interference with the addition of O-GlcNAc (1) results in elevation of UDP-GlcNAc and UDP-GalNAc (UDP-HexNAc) levels (2)**. Compensatory metabolic changes include increased flux into some complex glycans (3). Feedback inhibition of GFAT (4) is insufficient to fully normalize UDP-HexNAc levels in the absence of OGT-1. In addition, with increased nucleotide sugar pools, there are changes in upstream complex sugar levels (5) and decreased macronutrient storage (6). These changes in cellular nucleotide sugar pools are associated with altered transcriptional regulation of genes encoding hexosamine biosynthetic pathway and macronutrient storage enzymes (see text). Metabolite levels are increased (green), decreased (red), remain the same (gray), or were untested (black). Glc, glucose; G-6-P, glucose-6-phosphate; F-6-P, fructose-6-phosphate; GluN, glutamine; GlcN-6-P, glucosamine-6-phosphate; GlcNAc-6-P, *N*-acetylglucosamine-6-P; GlcNAc-1-P, *N*-acetylglucosamine-1-phosphate; acetyl-CoA, acetyl coenzyme A.

Cellular signaling depends on a series of protein posttranslational modifications working in concert to define protein localization, enzyme activity, and recognition events. Modifications ranging from glycosylation to phosphorylation are among the most widely recognized and defined posttranslational modifications with protein glycosylation being the most heterogeneous. The HBP produces a nutrient-sensitive nucleotide sugar, UDP-GlcNAc, that is utilized by glycosyltransferases in endo-membrane organelles (Golgi and endoplasmic reticulum) (23) as well as enzymes in the nucleus and cytoplasm (Figure 5). Optimal levels of UDP-GlcNAc are required for maintenance of cellular homeostasis in mammals with profound consequences including embryonic lethality resulting from loss of the nucleotide sugar synthesis (7, 24). The importance of this nucleotide sugar is evolutionarily conserved as loss or knockdown of enzymes required for synthesis of UDP-GlcNAc yields phenotypes including lethality in *C. elegans* (25–29).

Endo-membrane enzymes utilize UDP-GlcNAc to initiate N-glycosylation and further glycan branching (30). A portion of the UDP-GlcNAc pool is also used by OGT to glycosylate a myriad of nucleocytoplasmic targets ultimately influencing proteins’ localization, activity, and/or folding [see Ref. (8, 31) and references therein]. Perturbation of O-GlcNAc cycling yields a wide range of biological consequences; indeed, OGT and OGA are essential for viability in mice and other higher eukaryotes (11, 32). In viable *C. elegans* animals with loss-of-function of *ogt-1* and *oga-1* genes, we note altered carbohydrate and lipid metabolism as well as severely deregulated insulin signaling (9, 10, 33). Furthermore, we found that *C. elegans* animals with altered O-GlcNAc cycling have striking changes in fertility and reproductive timing during glucose stress suggesting that O-GlcNAc acts as a buffer to sense glucose availability (34). The work herein details the way in which perturbations in O-GlcNAc cycling influences changes in overall *C. elegans* carbohydrate composition as well as the transcription of key metabolic enzymes.

Our first efforts focused on identifying the ways in which changes to O-GlcNAc cycling affected nucleotide sugar concentrations and overall cellular carbohydrate structure. We noted that UDP-Glc and UDP-HexNAc (a combination of UDP-GlcNAc and UDP-GalNAc) were elevated 1.5- to 3-fold in the *ogt-1* and *oga-1* animals (Figure 1). Importantly, other nucleotide sugars such as GDP-mannose were not altered suggesting specific changes to the pool of activated sugars directly downstream of glucose metabolism. The elevation of UDP-Glc and UDP-HexNAc in both O-GlcNAc cycling mutants intrigued us. It is clear that in *ogt-1* loss-of-function animals, the UDP-GlcNAc pools would be elevated: the animals no longer utilize the activated nucleotide sugar to produce the O-GlcNAc PTM. Conversely, in animals that lack OGA-1 activity, while OGT-1 remains capable of utilizing UDP-GlcNAc to glycosylate protein substrates, the cycling of the PTM is likely altered and may influence the turnover of the nucleotide sugar pool. We speculate that this may explain the modest changes in nucleotide sugar concentrations and alterations in PNGAse-insensitive glycans but yield milder effects on the transcription of HBP members.

We hypothesized that among the consequences for even modest changes in activated nucleotide sugar pools, animals lacking *ogt-1* and *oga-1* would exhibit changes in N-glycan structures. Indeed, N-glycan structures have been shown to be ultrasensitive to UDP-GlcNAc concentration (19). We were, thus, surprised to find that N-glycan monosaccharide composition in both *ogt-1* and *oga-1* animals was indistinguishable from N2 animals (Figure 2A). These data suggest that the *C. elegans* mutants lacking O-GlcNAc cycling to have no global defect in constructing glycans sensitive to PNGase F and A. Interestingly, we found that glycans resistant to PNGase A or F (heterogeneous O-linked glycans, glycolipids, and glycan polymers) exhibited marked increases in galactose, GalNAc, and GlcNAc in both *ogt-1* and *oga-1* animals (Figure 2B). Recent work catalogs 14 types of mucin type O-linked glycans in the *C. elegans* WT strain with changes to O-linked glycans in another glycosyltransferase mutant (35). Future work will define the structural changes found in *ogt-1* and *oga-1* null animals’ PNGase resistant glycans. The compiled data suggest that the inability to add and remove O-GlcNAc on nuclear and cytoplasmic targets has far-reaching cellular affects affecting global glycosylation. These effects are likely to be pleiotropic and result from changes in transcription, metabolic flux, signaling, or direct enzyme activation. Among the reasons for these changes could be both direct and indirect perturbation of enzymes required for nucleotide sugar synthesis or the speed at which glycosylated proteins traffic through the secretory pathway (36).

With evidence suggesting that O-GlcNAc cycling is a key player in sensing cell nutrient status from our work and the work of others [see Ref. (31, 37) and references therein], we next hypothesized that the transcription of critical players in the HBP would be altered in the *ogt-1* and *oga-1* animals. The first, and rate limiting, enzyme of the HBP is GFAT, which is responsible for the conversion of fructose-6-phosphate to glucosamine-6-phosphate (Figure 5). To note, the activity of GFAT is modulated by UDP-GlcNAc itself through a feedback mechanism to reduce HBP flux (38). Two glucosamine-6-phosphate *N*-acetyltransferases (*gna-1* and *gna-2*) are required for an intermediary step in the HBP, and C36A4.4 is the presumptive pyrophosphorylase required for the last step in the synthesis of UDP-GlcNAc. We examined the expression levels for *gfat-2*, *gna-1*, *gna-2*, and C36A4.4 and noted that only in *ogt-1* mutants were there statistically significant changes in transcription for three of the four transcripts (Figure 3). Although feedback inhibition should occur in *ogt-1* animals due to increased UDP-HexNAc levels, it is insufficient to normalize the nucleotide sugar pool with increased *gfat-2* transcription. Moreover, these findings were surprising as PNGase-insensitive glycans were altered in both mutants suggesting a complex interplay between nutrient flux and appropriate substrate O-GlcNAc modification. (37).

Given that the O-GlcNAc cycling mutants exhibit variations in activated nucleotide sugar pools, changes in glycosylation (Figures 1 and 2), and increased glycogen and trehalose storage (9), we next assessed the levels of transcription for genes encoding enzymes involved in trehalose and glycogen metabolism. *C. elegans* encodes four putative glycoside hydrolases – enzymes responsible for catalyzing the conversion of trehalose to glucose – including *tre-1* and *tre-2*. *tps-2* encodes for one of two enzymes responsible for trehalose-6-phosphate synthesis and *gsy-1* is ortholog to the human glycogen synthase 1. All four of these transcripts were found to be elevated in *ogt-1* mutants while the levels remain unchanged in *oga-1* animals (Figure 4). These findings are consistent with our previous reports suggesting major changes in trehalose and glycogen metabolism upon genetic interference with O-GlcNAc cycling. The present findings suggest that these changes are associated with increased metabolic flux to produce UDP-Glc and with transcriptional changes in the transcripts encoding the relevant enzymes mediating interconversion.

Triglyceride levels have been shown to correlate with perturbations in O-GlcNAc cycling in both mice and *C. elegans* (9, 10, 21) and free fatty acids, usually derived from triglycerides or phospholipids, are known to be potent HBP modulators (39). Our previous work demonstrated that triglyceride levels are decreased by 70% in *ogt-1* and 40% in *oga-1* compared to N2 (9). These data suggest that either the production of triglycerides was hampered or their hydrolysis was increased. To test whether triglycerides are catabolized more rapidly in the O-GlcNAc cycling mutants, we assessed the transcriptional expression of Y73B6BL.4, a gene encoding for a phospholipase. We noted a significant increase in transcription for Y73B6BL.4 in both O-GlcNAc cycling mutants supporting that the decrease in triglyceride levels for both *ogt-1* and *oga-1* animals is likely associated with increased hydrolysis.

Perturbations of OGA activity in cell culture (40–42), loss of OGA-1 activity in *C. elegans* (9, 10), and OGT overexpression in mouse liver or fat promotes insulin resistance (43). Furthermore, loss of OGT-1 yields insulin sensitivity in *C. elegans* and altered lipid and carbohydrate metabolism (9, 10). Together, these data support a strong role for O-GlcNAc in insulin signaling metabolism maintenance. Our results reveal that loss of *ogt-1* and *oga-1* changes the nucleotide sugar pools and the production of PNGase-insensitive glycans. These changes along with altered transcriptional expression of genes encoding key HBP and metabolic enzymes in *ogt-1* null animals suggest that the addition of O-GlcNAc to appropriate target proteins is critical for appropriate HBP flux. Additional roles of OGT, including its non-catalytic role in protein–protein interactions (44), could also influence this signaling paradigm. We propose that with O-GlcNAc cycling profoundly affecting the HBP, *C. elegans* is an excellent model to studying metabolic changes associated with insulin signaling in viable *ogt-1* and *oga-1* null alleles (Figure 5). Using *C. elegans ogt-1* and *oga-1* animals, we will be able to further define the molecular details of the HBP’s role in insulin resistance.

## Conflict of Interest Statement

The authors declare that the research was conducted in the absence of any commercial or financial relationships that could be construed as a potential conflict of interest.
